# Window View and the Brain: Effects of Floor Level and Green Cover on the Alpha and Beta Rhythms in a Passive Exposure EEG Experiment

**DOI:** 10.3390/ijerph15112358

**Published:** 2018-10-25

**Authors:** Agnieszka Olszewska-Guizzo, Nicolas Escoffier, Jane Chan, Tan Puay Yok

**Affiliations:** 1 Department of Architecture, School of Design and Environment, National University of Singapore, Singapore 117566, Singapore; zcchan.jane@gmail.com (J.C.); akitpy@nus.edu.sg (T.P.Y.); 2 NeuroLandscape, 03-252 Warsaw, Poland; n.escoffier@neurolandscape.org; 3 Biomedical Institute for Global Health Research & Technology, National University of Singapore, Singapore 117599, Singapore

**Keywords:** window, view, landscape, high-rise, urban, brain, EEG

## Abstract

With the growing interest among researchers, practitioners, and urban decision makers in the influence of the built environment on peoples’ health, there is increasing emphasis on using scientific knowledge to inform urban design, including methods of neuroscience. As window views are the most immediate medium of visual connection with one’s neighbourhood, we surmised that the quality of this view would have an impact on the mental health and well-being of urban dwellers. Accordingly, we investigated how window views taken from different floors of a high-rise block with varying extents of green cover affected 29 healthy residents in an exploratory electroencephalography (EEG) experiment. The results showed that the amount of green cover within the view captured at different floor levels can cause an important interaction effect on the frontal alpha and temporal beta brain oscillations while participants view photographs. These results suggest that the brainwave patterns commonly associated with positive emotional states, motivation, and visual attention mechanisms may be increased by the extent of green cover within the view. This phenomenon seems more pronounced on the higher than lower floors. The observed findings at this stage cannot confirm major effects between floor level, green cover, and brainwaves, however, they emphasize the importance of considering the quality of window views in the planning and design of urban high-rise neighbourhoods. Having a green window view can potentially contribute to the mental health and well-being of urban dwellers.

## 1. Introduction

More than half the world’s population now lives in cities [1]. High population density often requires residential housing to go up, with increasing numbers of floors. In fact, going high-rise is a modern-day phenomenon, seen as a solution to the double conundrum of land scarcity and increasing numbers of urban dwellers, especially in populous Asian cities such as Singapore, Hong Kong, and Tokyo. 

Singapore is one of the most densely populated countries in the world, with over 7000 people per square km [2]. High-rise residential apartments, commonly called the Housing & Development Board (HDB) blocks, are ubiquitous, even iconic, in the Singapore landscape and are home to more than 80% of the population [2]. The newest HDB blocks stretch 50 floors and consist of 200 housing units on average. As living space rises further from the ground, direct physical contact with nature outdoors is often limited to moments when residents leave their apartments, take the elevator to the ground floor, and go outside. The only other possibility of contact with nature is visual access through apartment windows. 

### 1.1. Health and Well-Being of Residents of High-Rise Estates

Multiple studies in predominantly Western literature have shown reduced liveability of high-rise buildings as compared to ground floor living, especially for children [3,4,5,6]. High-rise estates are often associated with poor living conditions, including social isolation, segregation, pollution, and crime [7]. Critics cite problems of structural, internal and urban design, social, financial and management issues, etc. [8]. High-rise residential buildings are generally considered attributes of less liveable cities, as they do not have a human scale, separate people from the street life, and promote social isolation [9]. 

Interestingly, studies of Asia, where urbanization is occurring much more rapidly, take a different perspective, and scholars criticize the Western-oriented literature. For instance, Appold [10] observes that research on high-rise residential apartments tends to be skewed towards a selected segment of the population—the poor and working class—excluding the more affluent residents of such buildings. Because of critical blind spots caused by human biases towards the income status of the residents, studies tend to associate behaviours of poverty with the living situation. Upon closer study and controlling for confounding factors, Gifford [4] concludes that the commonly quoted negative effects can be eliminated when high-rise apartments are in better and more expensive neighbourhoods and residents can choose whether to stay there. This finding is supported by a nationwide survey conducted by the “Biophilic Town Framework” research project in Singapore; the project reveals that 74.9% of HDB residents are satisfied with their quality of life and 69.4% are satisfied with their health [11]. 

If we assume the high-rise environment has disadvantages, we should also assume the opposite is true: there are advantages. For example, a primary compensating factor for living in a high-rise environment is the potential to have a good view and be above one’s neighbours. Having a scenic view is widely known to add value to real estate, raising the price of houses in the United States by up to 90% and that of high-rise apartments in Singapore by 15% [12]. Even though the latter is smaller than the former, it is still notable. In Singapore, units on the upper floors of apartment buildings are generally priced higher than units on the lower floors. Similar effects are visible in Hong Kong, another high-rise city; here, proximity to neighbourhood parks can increase prices by 17%, with a 5% increase for a harbour view [13]. A caveat is in order: if high-rise buildings are located in high-density areas, window views may be blocked and higher floors may not confer higher prices. 

### 1.2. Psychological Responses to Urban Landscapes

The quality of window views plays an important role in the overall liveability of Singapore’s high-rise estates. It is the most immediate medium to visually connect residents with the outdoor environment. Studies in environmental psychology have firmly established the benefits of the presence of or interaction with green space or nature on human well-being [14,15]. Attention restoration theory (ART) suggests contact with the natural elements has a beneficial effect on stress reduction and recovery from mental fatigue [16]. In addition, Ulrich [17] demonstrates that hospital patients who have a window view over greenery recover faster and with less pain medication than patients without such a view. This phenomenon illustrates Wilson’s biophilia hypothesis [18], whereby humans have an innate desire to form connections with nature. 

Information about the surrounding environment is received through the five senses; vision is the dominant sense, estimated at providing 83% of information [19]. Visual sensory receptors capture information which is then transported through neurons to the brain where it is processed and interpreted. This suggests the value of looking at brain activity when we want to gauge the perception of environmental stimuli, a value heightened by the fact that during different activities and exposure to different stimuli, different parts of the brain are activated. 

The activity of the brain can be analysed through the electromagnetic waves it generates at different frequencies and amplitudes. These waves can be recorded using the method of electroencephalography (EEG). The four most important brainwave frequency bands are: (1) delta (<4 Hz); (2) theta (4–7.5 Hz); (3) alpha (7.5–14 Hz); and (4) beta (>14 Hz) [20]. They can be recorded using EEG as raw electrical signals from the brain in a laboratory environment. Research in psychology has established that specific brainwave patterns emerging from different areas of the brain can be related to different behaviours. Two categories of brainwaves are especially relevant to the present work. 

The first category of brain waves is alpha waves. Multiple studies have confirmed a relationship between increased right frontal alpha power and the human tendency to approach a stimulus perceived as positive or goal-relevant [21,22,23]. Beyond being an indicator of positive versus negative attitudes toward a stimulus, evidence suggests that this pattern of brain activity can be a marker of depression and other disorders affecting approach/withdrawal behaviour and motivation [24,25,26]. 

The second category of brainwaves is beta oscillations. Beta oscillations dominate in the waking state and have been associated with attentional processes, notably when they emerge from lateral and posterior parts of the brain [27,28]. These areas are involved in visual attention [29], interpretation of visual information and memory for pictures, visual scenes, and familiar faces [30,31]. This involvement of beta oscillations in visual processes and attention suggests that they are a potential marker for the attention mechanisms that support the shift from focused to involuntary attention that is proposed by ART.

While constantly evolving neuroscience provides new ways of assessing the impact of stimuli on mental health and well-being, studies considering the effect of green space or nature on the brain remain limited. An exception is a recent experiment testing the effect of walking through urban areas and green space, with subjects monitored by mobile EEG headsets [32]. A similar study has established that stress and anxiety are reduced when viewing external nature as compared to city scenes or indoor plants [33]. Other studies have assessed physiological responses to “forest bathing”, a therapy originating from Japan that emphasizes the healing effect of walking in a forest, and various exposures to urban vs. forest environments. These studies agree that exposure to a forest reduces anxiety and negative emotions, decreases the heart rate and salivary cortisol level, and increases positive emotions [34,35,36].

A recent study by the first author used the EEG method to test the possibility that exposure to certain physical attributes of urban parks and gardens can enhance well-being [37]. Participants’ brains responded differently to two blocks of stimuli identified as contemplative and noncontemplative landscape settings, presented through still-frame videos in a laboratory setting. The study used seven landscape features previously identified by a panel of experts as contributing to the level of contemplativeness: landscape layers, landform, vegetation, light and colour, compatibility, archetypal elements, and character of peace and silence. Differences were observed in the temporal beta and frontal alpha asymmetric oscillations. Given this set of results, it is possible that other conditions may elicit similar brain responses relating to human well-being.

### 1.3. Scope and Hypotheses

In this study, we investigated how the combinations of viewing height and the amount of green can impact the brainwaves of viewers. Findings have the potential to yield insights into how living in high-rise estates can influence the mental state of residents. Based on the literature reviewed above, we expected to see differences between the left and right brain hemisphere in alpha and beta responses when participants were shown different blocks of stimuli. We reasoned that by comparing responses from all blocks of stimuli, we might be able to pinpoint the optimal combination of floor level and green coverage. 

A particular focus of the study was the effects of the views on the power of the alpha oscillations in the frontal cortex (AF3, F3/AF4, F4). Increased right frontal alpha power was taken to indicate motivation, approach, and positive attitude toward the presented view, while increased left frontal alpha power was associated with withdrawal, avoidance, and negative attitudes. We hypothesized that:

**Hypothesis 1** **(H1).** An increasing level of greenery within the view would trigger greater right frontal alpha power reflecting a more positive response, a reaction anticipated by attention restoration theory (ART) or the biophilia hypothesis.

**Hypothesis 2** **(H2).** Greater right frontal alpha power would be associated with increasing floor level, given the arguments in the literature on the comfort of long-distance views and their benefits to mental health [38,39].

In addition, the study also examined the beta power band oscillations in the temporal region (T7/T8), giving rise to the third hypothesis:

**Hypothesis 3** **(H3).** Increased beta power would be seen in participants’ right temporal lobe when they were looking at pictures with higher levels of greenery and at a higher floor, reflecting the effect of these views on visual processes.

## 2. Materials and Methods

### 2.1. Participants

A total of 33 participants (14 female, 19 male) were recruited. Four data recordings were rejected because of excessive noise or movements, leaving the final number of participants as 29 (11 female, 18 male). The participants had to have resided in any HDB flat for more than a year, not necessarily the HDB flat from which the photographs were recorded. All participants were Singaporeans or Permanent Residents of Singapore, including Chinese (*n* = 19), Indians (*n* = 5), Eurasians (*n* = 4), and Malays (*n* = 1). Their ages ranged from 21 to 67 years (*M* = 31; *SD* = 10.3). The majority have a higher education of university degree and above (*n* = 22) or polytechnic diploma (*n* = 3). There were fewer participants with “O” Level (13–16 years old) education (*n* = 2) or “A” Level (17–18 years old) education (*n* = 2). 

The majority of the participants (*n* = 15) had lived in HDBs for over 20 years (between 21 and 52 years), fewer (*n* = 10) had lived there between 5 and 15 years, and a minority (*n* = 4) had lived there between 1 and 3 years. 

Most participants were right-handed; only three people reported left-handedness. All participants had normal or corrected to normal vision. Moreover, none reported a fear of heights and any psychiatric or neurological conditions, use of medication that could alter the functioning of the central nervous system at the time of the experiment. The existence of pacemakers, intracranial electrodes, implanted defibrillators or plates, otologic surgery in the last 12 months, or any dentures were additional exclusion criteria. None of the recruited participants was excluded.

All participants signed an informed consent form before participation. 

### 2.2. Materials

#### 2.2.1. Stimuli

**Figure 1:**
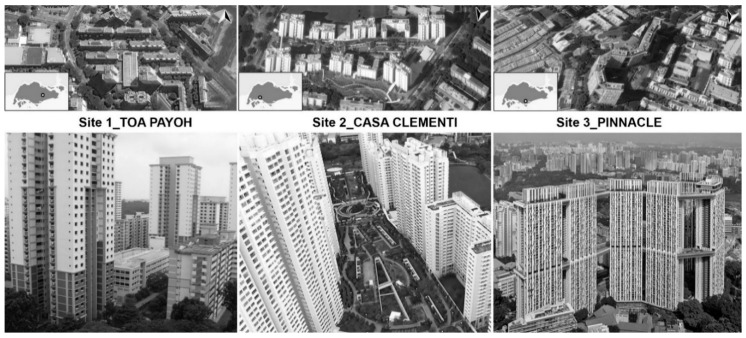
Location of three chosen sites for photo-stimuli around Singapore.

We decided to use photographs as a representation of the real window views in the laboratory setting as it poses no serious problems in terms of scientific validity. Studies show there is a strong positive correlation between the ratings of the photographs versus real landscape settings [40,41]. Thirty-six images representing window views from different floor levels and with different amounts of greenery within the view were prepared for the experiment. The preparation of the stimuli comprised two stages: Stage 1—visits to three selected HDB neighbourhoods (Pinaccle@Duxton, Toa Payoh, and Casa Clementi; see Figure 1), with photos collected from the 3rd, 6th, 12th, and 24th floor of the residential building. Stage 2—editing of the photographs to provide instances of three levels of green cover, minimal (11–20%), medium (28–41%), and high (50–78%), per view.

In Stage 2, these 12 photographs were processed with Autocad 2015 (Autodesk Inc., San Rafael, CA, USA) software to determine the percentage of green cover, then edited in Adobe PhotoShop CS4 (Adobe Systems Inc. San Jose, CA, USA,) to either increase or reduce the amount of green cover. The edited images were then assessed for the percentage of greenery within the image using Autocad 2015. 

**Figure 2:**
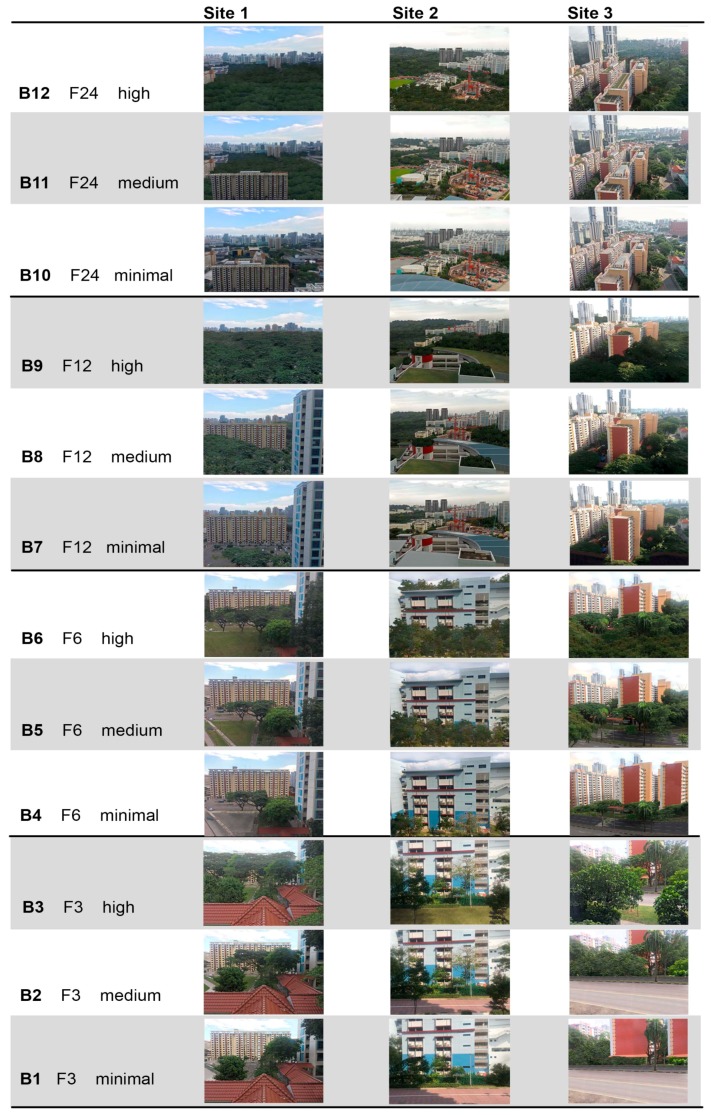
Photos of window views captured from three apartment buildings (Sites 1–3) and from four floor levels (F3—ground, 3rd floor, F6—6th floor, F12—12th floor, F24—24th floor) grouped into 12 blocks (B1–B12) and edited to fit into three green cover categories: minimal (<20%), medium (30–40%), and high (>50%).

Stage 1 yielded 12 site photographs, and this number was multiplied by three after Stage 2. The final set of 36 images was divided into 12 blocks of stimuli (B1–B12), each including three images, one from each site, with the same range of green cover and the same floor level (Figure 2). The mean pixel luminosity (lightness value in lab colour space) of all the images was between 110 and 150 units. All stimuli were presented in randomly ordered sequences using PsychoPy [42], where each of the 36 images was displayed for 10 s and preceded by a 2-s-long white fixation cross over a black background. Stimuli sequences were presented to each participant three times. 

**Figure 3:**
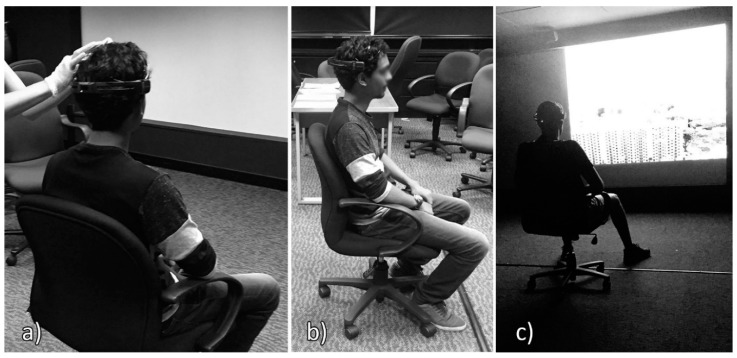
Experimental procedure: (**a**) adjusting the electrode placement, (**b**) participant ready for experiment, (**c**) presentation of stimuli while the electroencephalography (EEG) equipment acquires signal.

Total experiment duration per participant was 23 min. Images were presented using a NEC-M420X projector (NEC Display Solutions, Ltd., Tokyo, Japan) on a white screen (165 × 225 cm; see Figure 3c).

#### 2.2.2. EEG Apparatus

**Figure 4:**
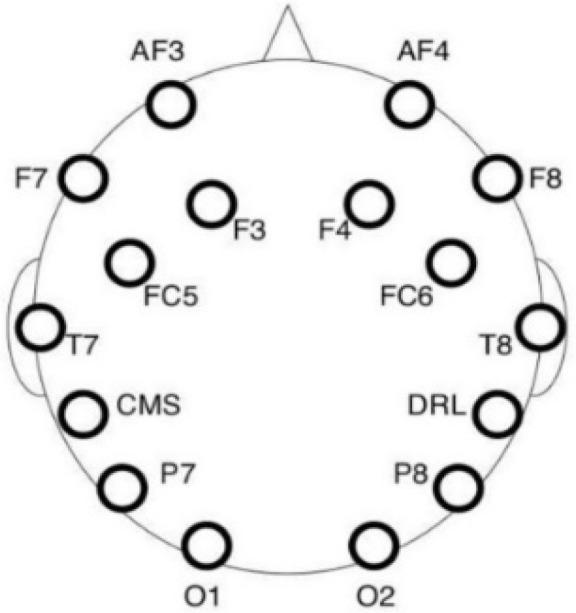
Emotiv electrode placement. The Emotiv Epoc headset is composed of 14 electrodes and 2 electrodes references (Source of image: Emotiv and Emotiv EPOC neuroheadset).

The EEG data were collected using a 16-channel electroencephalographic amplifier Emotiv EPOC+ (Emotiv Inc., San Fransicso, CA, USA). The montage consisted of 14 saline electrodes plus one Common Mode Sense (CMS) and one Driven Right Leg (DRL) electrode (P3/P4 locations respectively) arranged according to the 10–20 system, as depicted in Figure 4. The CMS electrode functioned as an implicit recording reference. The device was wireless (operating with Bluetooth) with a lithium-based battery and was connected to a laptop through the Emotiv Xavier Pure.EEG software Version 3.4.3 (Emotiv Inc., 2016). 

### 2.3. Procedure

#### Data Collection

Data acquisition took place in a quiet visualisation lab on the university premises. All electronic devices in the room were switched off, except the stimulation and EEG recording systems, to reduce all possible external electromagnetic artefacts. Subjects were instructed to fill out the informed consent and sociodemographic questionnaire prior to the experiment. In the latter, they were asked, among the general information, to provide information on which floor they live on, what they could see from their windows, and if they were afraid of heights. During the experiment, participants were instructed to passively observe all images of the window views. 

Baseline resting state was recorded for 2 min (1 min of eyes closed and 1 min of eyes opened) before the experiment start. These data were not the focus of the present study and were not examined further. During the experiment, the stimulation software sent event markers to the acquisition software through a virtual port as each image came on screen. 

The scalp of the participant was cleaned with a cotton pad wetted with ethylic alcohol, with special attention to the areas where the electrodes were placed. The headset with 14 active electrodes was placed on the participant’s head, and the embedded CMS/DRL electrodes were located at the P3/P4 locations (see Figure 3). Electrode impedance was decreased by using saline liquid until the level required by the software was reached (in the 10–20 kΩ range). The EEG signal was acquired at a 256 Hz sampling rate.

Participants were seated on a chair placed 2.5 m in front of the projection wall. Those with correction lenses kept their glasses on. Participants were instructed to watch the window views and imagine they were looking out a window. Each participant was informed about the two parts of the experiment, baseline recording and window view presentation, and the duration of each. 

### 2.4. Data Processing and Analysis

The EEG data were processed with the EEGLAB toolbox (version 14.1.1b [43]) in Matlab (version 7.11.0.584, The Mathworks Inc., Natick, MA, USA). Raw data were imported from Emotiv software text output and filtered using high-pass sync filters (stop band edge: 3 Hz, 425 points, transition band: 2 Hz). Artefactual sections of the data were interpolated using the Artifact Subspace Reconstruction technique in EEGLAB ([44]; burst criterion: 20 SDs, Window criterion: 0.75), and noisy channels were removed using the random sample consensus method (RANSAC [45]; criterion: R < 0.80). The resulting signal was visually examined, and participants with excessive residual movement and muscle artefacts were rejected. Data then underwent an independent component analysis (ICA; [46]), after which we visually identified and rejected components that captured eye movement, eye blinks, and cardiac artefacts. Data were back-projected to yield an artefact-free EEG signal, and previously discarded channels were replaced using spherical interpolation. The EEG signal was cut into epochs time-locked to stimulus onset; each had a 2-s prestimulus baseline and a 10.5-s poststimulus interval. EEG epochs were baseline corrected and re-referenced to a 14-channel average reference. An average reference was chosen because it is standard for EEG oscillations analysis [47], and it allowed to overcome the lateralization bias introduced by the left placement of the implicit CMS recording reference. 

EEG epochs were decomposed into frequency bands of interest using a continuous wavelet transform implemented in EEGLAB [31]. Wavelet decomposition covered frequencies from 6 to 20 Hz (*c* = 3–10, increasing linearly). This covered the frequency bands of interest: alpha (8–13) and beta (14–20). Wavelet output was used to compute raw power, and data were normalized using a full epoch average, followed by a normalization using average baseline power taken between −500 and −250 ms before image onset [48]. The baseline interval did not extend until onset, thus preventing responses close to image onset from influencing baseline measurements through smearing [7]. Data were transformed to a decibel scale (10*log (signal) and averaged over frequency bands of interest for further analysis. Because we were interested in the sustained responses to images, we examined power averages over a 1–9 s interval postimage onset.

#### Statistical Analysis

EEG power was analysed for each frequency band and electrode group of interest separately using three-way repeated measures ANOVAs with three factors: Green cover (3 levels), Floor (1st, 6th, 12th, 24th), and Hemisphere (left and right). To explore differences between specific factor cell mean, we performed post hoc pairwise analysis and controlled for multiple comparisons (Holm–Sidak method). For each ANOVA, we reported generalized eta square effect sizes. In addition, to assess the robustness of the results, pairwise comparisons of interest were further investigated using a Bayesian probabilistic approach. This has several advantages over traditional *t*-tests, notably estimates are more robust to the presence of outliers and require less assumptions on the nature of the data (see [49] for further details). For each comparison of interest, we computed the probability that block differences were in the observed direction. Probabilities were computed in R (http://www.r-project.org/) from the posterior distribution computed using Markov chain Monte Carlo (MCMC) sampling performed in JAGS [50].

## 3. Results

### 3.1. Mean Frontal Alpha Power (AF3, F3/AF4, F4)

**Figure 5:**
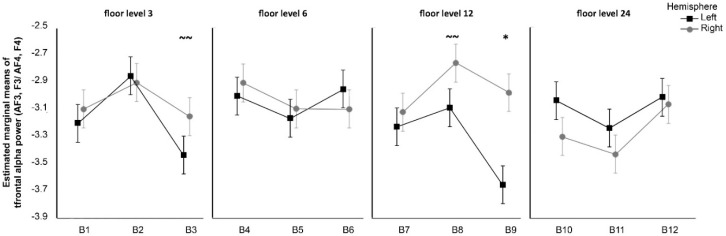
Differences in mean values between Hemisphere and Block for different floors. Significant differences (*p* < 0.05): asterisks (*), trend towards significance (0.10 < *p* < 0.20): double tilde (~~). Error bars represent within subject standard error of the mean [51].

The three-way repeated measures ANOVA revealed that for the alpha frontal oscillation, there was a marginally significant interaction between the factors Green cover, Floor, and Hemisphere *F*(4,112) = 1.98, *p* = 0.10, *η²* = 0.002. No other effects were significant (*p*s > 0.48). Follow-up of the three-way interaction using pairwise multiple comparison procedures revealed a significant difference between hemispheres within B9 (*p* = 0.002) with the Bayesian approach, indicating a 99.96% probability of greater power in the right hemisphere. A trend toward significance was observed within B8 (*p* = 0.123), with 89.2% probability of greater power in the right hemisphere, and within B3 (*p* = 0.185), with 92.98% probability of greater power in the right hemisphere. No other pairwise block differences were significant or showed a trend towards significance (*p* > 0.214) (Figure 5).

To sum up, there was significantly greater right frontal alpha power in participants exposed to photos taken from the 12th floor with the highest amount of green cover. For the photos with highest or medium green cover taken from other floors, the results showed a trend towards significance within the 3rd and 12th floors, but no significance was observed in the case of 6th and 24th floors.

### 3.2. Mean Temporal Beta Power (T7/T8)

**Figure 6:**
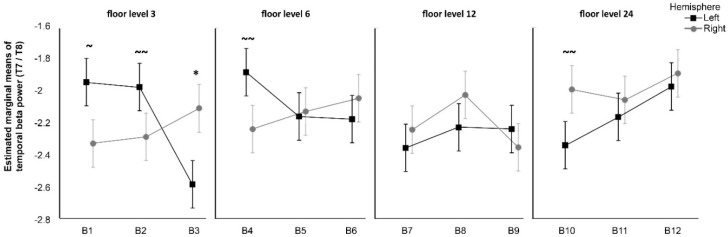
Differences in mean values between Hemisphere and Block for different floors. Significant differences (*p* < 0.05): asterisks (*), marginally significant (0.05 < *p* < 0.10): tilde (~), trend towards significance (0.10 < *p* < 0.20): double tilde (~~). Error bars represent within subject standard error of the mean [51].

The three-way repeated measures ANOVA revealed a significant interaction between the factors Green cover, Floor, and Hemisphere *F*(4,112) = 2.97, *p* = 0.022, *η²* = 0.01. No other effects were significant (*p*s > 0.37). Follow-up of the three-way interaction using pairwise multiple comparison procedures revealed a significant difference between hemispheres for B3 (*p* = 0.033), with Bayesian analysis indicating 95.91% probability of greater power in the right hemisphere. There was a trend toward significance for the same effect in B10 (*p* = 0.118), with 89.7% probability of greater power in the right hemisphere. The opposite difference between hemispheres was marginally significant for B1 (*p* = 0.087), with 94.1% probability of greater power in the left hemisphere, and there was a trend toward significance for B2 and B4 (resp., *p* = 0.159 and *p* = 0.112), with resp. 92.6% and 94.4% probability of greater power in the left hemisphere. No other differences were significant (*p* > 0.356) (Figure 6). 

Summing up, the pattern of greater right beta temporal activity as compared to the left was significant only in the case of the highest level of green on the 3rd floor. Moreover, the trend towards significance was observed within the least-green views from the 24th floor. The opposite brainwave pattern, namely, the greater beta temporal on the left than on the right side of the brain, was observed as a trend towards significance, for the lowest and medium green covers within the view in the case of 3rd and 6th floors.

## 4. Discussion

The objective of this study was to explore if the conditions of height of view and amount of vegetation have an effect on brain responses and whether a certain combination is beneficial to well-being in a high-rise environment. The study has two main findings. 

First, the analysis of frontal alpha oscillations revealed significantly greater right frontal alpha power in participants exposed to photos taken from the 12th floor with the highest amount of green cover. In light of the approach-withdrawal hypothesis [23], this suggests that participants displayed approach behavioural tendencies towards these views, likely related to associated positive attitudes. Another possibility is that participants liked those scenes or had feelings of pleasure when they appeared, following the findings of Davidson et al. [21], who examined brainwaves of participants simultaneously watching and evaluating television shows. Positively rated TV scenes were associated with greater relative left-hemispheric alpha frontal activation, while negatively rated scenes were associated with greater relative right-hemispheric frontal activation. 

Given the above findings, the first hypothesis (H1) was not fully supported—we observed that only the 12th floor, greenest window view induced increased right frontal alpha power (we did not observe a significant effect for the 3rd, 6th, and 24th floors). However, these results may indicate that the level of green cover within a view may cause an important interaction in increasing positive motivation and approach and/or decreasing the withdrawal response in the participants. Of course, the brain responses of participants might indicate differences in underlying affective responses, as alpha frontal activation is related to joy and relaxation or disgust and fear [22], but we did not perform an affective study nor a study on the levels of familiarity of participants with the perceived stimuli. This remains for future work. 

The floor level did not seem to play a role in the alpha frontal oscillations, although views from the higher floors may have induced a withdrawal effect in the participants. Therefore, the second hypothesis (H2) was not substantiated.

Second, the result of the analysis of beta temporal oscillations suggest an interaction between certain window views and attention mechanisms; this might be related to spatial recognition and the ability to grasp the entire image instead of its fragments. EEG studies show that the attention shift mechanism is associated with increased temporal beta in the right compared to the left hemisphere [28,52]. A similar pattern appeared in Olszewska’s [37] study, in which it was found that when participants were observing 3D videos of landscapes classified as the most contemplative, their right temporal brain was more engaged as compared to when they were observing noncontemplative images. In the current study, this pattern occurred with significance only for the ground floor photos with maximal levels of green cover. A trend for the same effect was suggested for the 24th floor photos with minimal green cover. This corresponds to earlier mentioned benefits of interaction with nature as demonstrated in Ulrich’s [17] window view study, that is reinforced by Kaplan’s [16] attention restoration theory and Wilson’s [18] biophilia hypothesis. 

Decreased temporal beta power effect, although not significant, was suggested for blocks at lower floors and higher levels of green cover. While we must be cautious in interpreting these patterns, it is possible that the proximity of lush green observed from the lower floor enhanced focused visual attention because of the relatively easy access to an outdoor landscape and familiarity of the element of nature. A reverse effect was observed for photographs with lower green cover taken from the lower floors, suggesting that less-green window views may not induce a positive motivation. Nevertheless, the collected evidence is not sufficient to support the third hypothesis (H3) for all investigated instances but may suggest important interaction effects that may be explored in future studies. 

Interestingly, beta oscillations effects showed a trend towards significance within B10, the least-green view from the 24th floor. This finding seems contradictory given the above analysis. It may reflect a complex phenomenon occurring in the brain—an increased beta temporal activity in the right hemisphere ought to be juxtaposed to less alpha power in that hemisphere. The visual attention, combined with the pattern of withdrawal, may be then interpreted as a negative phenomenon. This can possibly relate to a natural reaction (i.e., cautiousness) in regard to height as objects on the ground get smaller and become less human scale in the eye of the viewer. 

The approach we used for this study was exploratory. It was meant as a first step toward future research that would confirm these findings and reach stronger conclusions. Limitations include the use of relatively noise-sensitive equipment for EEG recording. The sample size was limited and was adequate to detect medium-to-major effects only. We did not include a large number of conditions (floors), and this limits our ability to explore the continuity of observed phenomena. Further research should address these limitations.

Nevertheless, this study shows that different amounts of greenery within a window view can alter the brainwave patterns of the observer. Higher levels of green coverage within the window view can induce brainwave patterns associated with approach, motivation, and relaxation. They may have the potential to help with certain mental health disorders. The expectation of more positive brain responses from higher floors appears less accurate, as the pattern of positive approach seemed to reverse between the 12th and 24th floors. Window views from the highest floors may stimulate people’s brains in a less positive way, but an increased level of green cover within the view may potentially limit this effect.

## 5. Conclusions

To the best of our knowledge, this is the first experimental study to assess the effects of the combination of floor level and green cover within a window view on the brainwave oscillations of healthy individuals living in high-rise estates. 

The experiment emphasizes the importance of the quality of window views as the most immediate contact with the outdoors for urban dwellers and validates their potential interaction with well-being. The design of window views should be considered by urban planners and architects in new high-rise buildings. A preliminary design recommendation is that very tall buildings, generally over 24 floors, may not be beneficial to residents in terms of mental health and well-being, although negative effects can be mitigated with increased green coverage visible from the higher floors, underlining the importance of tree canopies. 

To the increasing scientific evidence of the benefits of nature on people, extending beyond physical health, this study provides a supplementary idea: landscape design can be considered from the perspective of the window view. 

As cities focus on urban greening strategies to increase the provision of nature to their inhabitants, findings from this study take on added significance. Future research would be well-advised to continue to investigate the relationship between the features present in the window views of high-rise apartments and the brain responses of healthy individuals, as these features may be directly related to mental health and well-being.

## References

[1] United Nations, Department of Economic and Social Affairs (2014). World Urbanization Prospects: The 2014 Revision, Highlights.

[2] Singapore Department of Statistics (DOS). Singstat.gov.sg.

[3] Gillis A. (1977). High-Rise Housing and Psychological Strain. J. Health Soc. Behav..

[4] Gifford R. (2007). The Consequences of Living in High-Rise Buildings. Archit. Sci. Rev..

[5] Kearns A., Whitley E., Mason P., Bond L. (2012). ‘Living the high life’? Residential, social and psychosocial outcomes for high-rise occupants in a deprived context. Hous. Stud..

[6] Oda M., Taniguchi K., Wen M.Z., Higurashi M. (1989). Symposium: Environment and Human Behavior (II) Effects of High-rise Living on Physical and Mental Development of Children. J. Hum. Ergol..

[7] Helleman G., Wassenberg F. (2004). The renewal of what was tomorrow’s idealistic city. Amsterdam’s Bijlmermeer high-rise. Cities.

[8] Turkington R., Van Kempen R., Wassenberg F. (2004). High-rise housing in Europe: Current trends and future prospects. Housing and Urban Policy Studies.

[9] Cappon D. (1971). Mental health in the high-rise. Can. J. Public Health/Revue Canadienne de Sante’e Publique.

[10] Appold S., Yuen B., Yeh A. (2017). Community Development in Tall Residential Buildings. In the March of High-Rise.

[11] Tan P.Y., Liao K.H., Hwang Y.H., Chua V. (2018). Nature, Place & People. Forging Connections through Neighbourhood Landscape Design.

[12] Lothian A. (2013). The Science of Scenery: How We See Scenic Beauty, What It Is, Why We Love It, and How to Measure and Map It.

[13] Jim C., Chen W. (2010). External effects of neighbourhood parks and landscape elements on high-rise residential value. Land Use Policy.

[14] Kuo F., Bacaicoa M., Sullivan W. (1998). Transforming Inner-City Landscapes. Environ. Behav..

[15] Louv R. (2008). Last Child in the Woods: Saving Our Children from Nature-Deficit Disorder.

[16] Kaplan R., Kaplan S. (1989). The Experience of Nature.

[17] Ulrich R. (1984). View through a window may influence recovery from surgery. Science.

[18] Wilson E. (1984). Biophilia.

[19] Medina J. (2014). Brain Rules.

[20] Niedermeyer E., da Silva F.L. (1993). Electroencephalography: Basic Principles, Clinical Applications, and Related Fields.

[21] Davidson R.J., Schwartz G.E., Saron C., Bennett J., Goleman D.J. (1979). Frontal versus parietal EEG asymmetry during positive and negative affect. Psychophysiology.

[22] Davidson R., Hugdahl K. (1998). Brain Asymmetry.

[23] Harmon-Jones E., Gable P., Peterson C.K. (2010). The role of asymmetric frontal cortical activity in emotion-related phenomena: A review and update. Biol. Psychol..

[24] Baehr E., Rosenfeld J., Baehr R. (1997). The Clinical Use of An Alpha Asymmetry Protocol in the Neurofeedback Treatment of Depression. J. Neurother..

[25] Rosenfeld J., Cha G., Blair T., Gotlib I. (1995). Operant (biofeedback) control of left-right frontal alpha power differences: Potential neurotherapy for affective disorders. Biofeedback Self-Regul..

[26] Spronk D., Arns M., Bootsma A., van Ruth R., Fitzgerald P. (2008). Long Term Effects of Left Frontal rTMS on EEG and ERPs in Patients with Depression. Clin. EEG Neurosci..

[27] Escoffier N., Herrmann C.S., Schirmer A. (2015). Auditory rhythms entrain visual processes in the human brain: Evidence from evoked oscillations and event-related potentials. NeuroImage.

[28] Wróbel A. (2000). Beta activity: A carrier for visual attention. Acta Neurobiol. Exp..

[29] Kolb B., Whishaw I. (1990). Fundamentals of Human Neuropsychology.

[30] Milner B. (1968). Visual recognition and recall after right temporal-lobe excision in man. Neuropsychologia.

[31] Tallon-Baudry C., Kreiter A., Bertrand O. (1999). Sustained and transient oscillatory responses in the gamma and beta bands in a visual short-term memory task in humans. Vis. Neurosci..

[32] Neale C., Aspinall P., Roe J., Tilley S., Mavros P., Cinderby S. (2017). The Aging Urban Brain: Analyzing Outdoor Physical Activity Using the Emotiv Affectiv Suite in Older People. J. Urban Health.

[33] Chang C.Y., Chen P.K. (2005). Human response to window views and indoor plants in the workplace. HortScience.

[34] Kobayashi H., Song C., Ikei H., Kagawa T., Miyazaki Y. (2015). Analysis of Individual Variations in Autonomic Responses to Urban and Forest Environments. Evid.-Based Complement. Alternat. Med..

[35] Ochiai H., Ikei H., Song C., Kobayashi M., Takamatsu A., Miura T. (2015). Physiological and Psychological Effects of Forest Therapy on Middle-Aged Males with High-Normal Blood Pressure. Int. J. Environ. Res. Public Health.

[36] Song C., Ikei H., Miyazaki Y. (2016). Physiological Effects of Nature Therapy: A Review of the Research in Japan. Int. J. Environ. Res. Public Health.

[37] Olszewska-Guizzo A.A., Paiva T.O., Barbosa F. (2018). Effects of 3D Contemplative Landscape Videos on Brain Activity in a Passive Exposure EEG Experiment. Front. Psychiatry.

[38] Skalski J. (2005). Comfort of Long-Distance Perceiving and a Landscape of River Valley in Towns Situated on the Plains. Teka Komisji Architektury, Urbanistyki i Studiów Krajobrazowych.

[39] Tuan Y. (1990). Topophilia.

[40] Pitt D., Zube E.H., Stokols D., Altman I. (1987). Management of Natural Resources. Handbook of Environmental Psychology.

[41] Stamps A.E. (1990). Use of photographs to simulate environments: A meta-analysis. Percept. Motor Skills.

[42] Peirce J. (2007). PsychoPy—Psychophysics software in Python. J. Neurosci. Methods.

[43] Delorme A., Makeig S. (2004). EEGLAB: An open source toolbox for analysis of single-trial EEG dynamics including independent component analysis. J. Neurosci. Methods.

[44] Mullen T., Kothe C., Chi Y., Ojeda A., Kerth T., Makeig S. (2015). Real-time neuroimaging and cognitive monitoring using wearable dry EEG. IEEE Trans. Biomed. Eng..

[45] Fischler M., Bolles R. (1981). Random sample consensus: A paradigm for model fitting with applications to image analysis and automated cartography. Commun. ACM.

[46] Jung T., Makeig S., Westerfield M., Townsend J., Courchesne E., Sejnowski T. (2000). Removal of eye activity artifacts from visual event-related potentials in normal and clinical subjects. Clin. Neurophysiol..

[47] Cohen M.X. (2014). Analyzing Neural Time Series Data: Theory and Practice.

[48] Grandchamp R., Delorme A. (2011). Single-Trial Normalization for Event-Related Spectral Decomposition Reduces Sensitivity to Noisy Trials. Front. Psychol..

[49] Kruschke J.K. (2013). Bayesian estimation supersedes the t test. J. Exp. Psychol..

[50] Plummer M. JAGS: A program for analysis of Bayesian graphical models using Gibbs sampling. Proceedings of the 3rd International Workshop on Distributed Statistical Computing.

[51] Masson M., Loftus G. (2013). Using confidence intervals for graphically based data interpretation. Can. J. Exp. Psychol..

[52] Fink G. (1997). Neural mechanisms involved in the processing of global and local aspects of hierarchically organized visual stimuli. Brain.

